# The effects of combined magnesium and zinc supplementation on metabolic status in patients with type 2 diabetes mellitus and coronary heart disease

**DOI:** 10.1186/s12944-020-01298-4

**Published:** 2020-05-28

**Authors:** Zahra Hamedifard, Alireza Farrokhian, Željko Reiner, Fereshteh Bahmani, Zatollah Asemi, Maryam Ghotbi, Mohsen Taghizadeh

**Affiliations:** 1 Research Center for Biochemistry and Nutrition in Metabolic Diseases, Institute for Basic Sciences, Kashan University of Medical Sciences, Kashan, Iran; 2 Department of Cardiology, School of Medicine, Kashan University of Medical Sciences, Kashan, Iran; 3 Department of Internal Medicine, University Hospital Centre Zagreb, School of Medicine, University of Zagreb, Zagreb, Croatia

**Keywords:** Coronary heart disease, Magnesium, Metabolic status, Type 2 diabetes mellitus, Zinc

## Abstract

**Background:**

The present research aimed to analyze the impacts of magnesium and zinc supplements on glycemic control, serum lipids, and biomarkers of oxidative stress and inflammation in patients suffering from coronary heart disease (CHD) and type 2 diabetes mellitus (T2DM).

**Methods:**

According to the research design, a randomized, double-blind, placebo-controlled trial has been implemented on 60 subjects suffering from CHD and T2DM. Therefore, participants have been randomly divided into 2 groups for taking placebo (*n* = 30) or 250 mg magnesium oxide plus 150 mg zinc sulfate (*n* = 30) for 12 weeks.

**Results:**

Magnesium and zinc significantly decreased fasting plasma glucose (FPG) (β − 9.44 mg/dL, 95% CI, − 18.30, − 0.57; *P* = 0.03) and insulin levels (β − 1.37 μIU/mL, 95% CI, − 2.57, − 0.18; *P* = 0.02). Moreover, HDL-cholesterol levels significantly enhanced (β 2.09 mg/dL, 95% CI, 0.05, 4.13; *P* = 0.04) in comparison to the placebo. There was an association between magnesium and zinc intake, and a significant decrease of C-reactive protein (CRP) (β − 0.85 mg/L, 95% CI, − 1.26, − 0.45; *P* < 0.001), a significant increase in total nitrite (β 5.13 μmol/L, 95% CI, 1.85, 8.41; *P* = 0.003) and total antioxidant capacity (TAC) (β 43.44 mmol/L, 95% CI, 3.39, 83.50; *P* = 0.03) when compared with placebo. Furthermore, magnesium and zinc significantly reduced the Beck Depression Inventory index (BDI) (β − 1.66; 95% CI, − 3.32, − 0.009; *P* = 0.04) and Beck Anxiety Inventory (BAI) (β − 1.30; 95% CI, − 2.43, − 0.16; *P* = 0.02) when compared with the placebo.

**Conclusions:**

In patients with T2DM and CHD, the 12-week intake of magnesium plus zinc had beneficial effects on FPG, HDL-cholesterol, CRP, insulin, total nitrite, TAC levels, and BDI and BAI score. This suggests that magnesium and zinc co-supplementation may be beneficial for patients with T2DM and CHD. Further studies on more patients and lasting longer are needed to determine the safety of magnesium and zinc co-supplementation.

**Trial registration:**

Current Controlled Trials http://www.irct.ir: IRCT20130211012438N31 at 11 May 2019 of registration. This study retrospectively registered.

## Background

As reported by the World Health Organization (WHO), coronary heart disease (CHD) is a primary cause of both men and women’s mortality, which results in more than 7 million deaths annually [1]. It is widely accepted that type 2 diabetes mellitus (T2DM) is also one of the important risk factors for CHD. The risk of CHD in patients with diabetes is double as high as in non-diabetic subjects [2]. Many studies have shown that there is a correlation between CHD and metabolic syndrome (MetS), T2DM, and elevated levels of inflammatory and oxidative stress biomarkers [3, 4]. Some researches indicated that levels of magnesium and zinc are significantly decreased in patients suffering from CHD and T2DM [5, 6].

It has been already reported that there are beneficial impacts of trace elements on the metabolic profile in the patients suffering from metabolic disorders [7–9]. For example, Asemi et al. showed that magnesium supplementation with a dose of 250 mg per day in the form of magnesium oxide to the pregnant females suffering from gestational diabetes (GDM) considerably improved glycemic control and lipoproteins as well as oxidative stress and inflammation biomarkers [7]. Zinc supplementation at a dose of 30 mg per day in the form of zinc sulfate by subjects with pre-diabetes for 6 months caused a significant improvement in glycemic control [10]. It was reported that co-supplementation is possibly more effective when compared to single element supplementation [11]. A recent study on patients with T2DM indicated that zinc, magnesium, vitamin C and E co-supplementation significantly reduced fasting plasma glucose (FPG) and malondialdehyde (MDA) levels and considerably increased the levels of HDL-cholesterol [12]. Results also showed that zinc and magnesium co-supplementation to females suffering from polycystic ovary syndrome (PCOS) improved high sensitivity C-reactive protein (hs-CRP) as well as total antioxidant capacity (TAC); however, it had no effect on the other markers of oxidative stress [13]. Magnesium-zinc-calcium-vitamin D co-supplementation in the patients with PCOS showed that there is an association between such a co-supplementation and a significant improvement in insulin levels, quantitative insulin sensitiveness check index (QUICKI), inflammatory markers, decrease in plasma triglycerides, total cholesterol and VLDL-cholesterol and homeostatic model of insulin resistance (HOMA-IR), without any significant influence on the level of fasting glucose, HDL-cholesterol, and LDL-cholesterol [14].

Both zinc and magnesium contribute substantially to the glucose homeostasis and lipoprotein metabolism. Magnesium participates as a cofactor in different adenosine triphosphate depended reactions which are important in carbohydrate metabolism and insulin action [15]. Magnesium also contributes to lipoprotein metabolism by modulating the 3-hydroxy-3-methyl-glutaryl-CoA (HMG-CoA) reductase enzyme [16]. Some studies also demonstrated that zinc affected glucose homeostasis in terms of formation, storage, and secretion of insulin [17–20]. It seems that magnesium could have anti-inflammatory properties having antagonist effects to calcium which contributes significantly to the inflammation, transmembrane ion transport, and protein synthesis [21] as well as increasing production of prostacyclins [22]. Moreover, zinc can decrease inflammation and oxidative damage having beneficial effects on hemostasis by influencing coagulation and platelet accumulation [23], and reducing the activity of calcium channels. Also, calcium uptake defect and impaired second-messenger function results from an abnormal sulfhydryl redox state in the membrane channel protein may have an impact on cardiovascular disease (CVD) [24].

There is a lot of literature focused on the effects of co-administration of zinc and magnesium in patients with CHD or T2DM and the management of insulin resistance, serum lipoproteins, oxidative damage and inflammation in these patients. No study so far has evaluated the role of zinc and magnesium co-supplementation in patients with CHD and T2DM and there is insufficient evidence to recommend such a combined supplementation for these patients. Therefore, the aim of this study was to analyze the effects of combination of magnesium and zinc supplementation on inflammation biomarkers, metabolic profile, and oxidative stress but also some mental health parameters in patients suffering from CHD and T2DM.

## Methods

### Study population

This was a double-blind, randomized placebo-controlled research, which was registered in the Iranian registry of clinical trials at http://www.irct.ir: no, IRCT20130211012438N31. This research was done at the cardiology clinic, which is affiliated to Kashan University of Medical Sciences (KAUMS), Kashan, and Iran. It lasted from January 2019 to May 2019. The study was performed following the Declaration of Helsinki principles. The research design was approved by the Research Ethics Committee of KAUMS (no. IR.KAUMS.MEDNT.REC.1397.079), Iran. Each patient signed a written informed consent. The criteria for including in the study were: patients suffering from T2DM in the age ranging between 40 and 95 years with proven 2- and 3-vessel CHD, and no smoking. T2DM and CHD diagnosis was made based upon American Diabetes Association criteria [25] and the American Heart Association criteria [26]. Some patients were excluded based on the following criteria: taking any type of supplements such as magnesium and/or zinc 3 months before the study, consuming antioxidant and or anti-inflammatory supplements and/or omega-3 fatty acids, proven renal or hepatic failure, acute myocardial infarction, or cardiac surgery in the last 3 months, thyroid disease, any change in LDL levels after the 6-week intervention, infection, unwillingness to cooperate, and antibiotic use during study.

### Research design

Patients were randomly assigned into 2 treatment groups (after strafication based on baseline BMI and age): one taking 250 mg per day magnesium (magnesium oxide) and 150 mg/day zinc (zinc sulfate) containing 30 mg elementary zinc or placebo (starch) (Barij Essence; Kashan: Iran) (*n* = 30 in each group) for 12 weeks. Due to the lack of evidence about the appropriate dosage and duration of zinc and magnesium co-supplementation for subjects with T2DM and CHD, the above-mentioned dose and duration of zinc and magnesium used based on previous studies in patients with PCOS [13] and pre-diabetes [10]. Color, form, size, and package of the placebo and magnesium plus zinc supplements were similar. Randomizing has been done by computer-generated random numbers. The investigators and patients have been blinded concerning the randomization and supplements/placebo until the final analyses have been made. Enrolling the patients, randomizing, and assigning them to the treatment or placebo have been performed by the qualified personnel at the clinic. Compliance with taking placebo and the supplement has been made by examining the capsule containers. Each patient filled in the three-day dietary intake record at 1, 6, and 12 weeks of the trial. The dietary records were filled in according to estimated values household measurements. Nutritionist IV software (First Databank; San Bruno; CA) adapted for the Iranian food pattern has been employed to achieve the patients’ nutrient intakes in accordance with three-day food records. Patients weight (using Seca; Hamburg: Germany balance) to the nearest 0.1 kg and height (using Seca; Hamburg: Germany) to the nearest 0.1 cm were measured at baseline and 12 weeks after clinical intervention. BMI was calculated. All anthropometric measurements were done by a qualified nutritionist.

### Assessment of biochemical variables

The primary outcome was insulin resistance and other metabolic parameters were secondary outcomes.

According to research design, fasting blood samples (10 mL) have been drawn out at the baseline and after a 12-week period of intervention at Kashan Reference Laboratory. Blood was collected in 2 separate tubes: 1) one without EDTA and trace mineral free to separate the serum and to quantify serum magnesium, zinc, insulin, lipids and CRP and 2) another containing EDTA to measure plasma total nitrite and biomarkers of oxidative stress. Serum insulin was assessed using ELISA kit (DiaMetra; DKO076 code, Milano: Italy) through inter- and intra-assay coefficient variances (CVs) beneath 5%. QUICKI and HOMA-IR were evaluated on the basis of the standard formulation [27]. Enzymatic kits (Pars Azmun; Tehran: Iran) were used to estimate serum magnesium, zinc, FPG, serum lipoproteins that had inter-assay and intra-assay CVs beneath 5%. CRP levels were evaluated using an ELISA kit (LDN; DM E-4600 Tags code, Nordhorn: Germany) with inter-assay and intra-assay CVs below 7%. Total nitrite were determined by Griess assay [28] and TAC by the technique published by Benzie and Strain [29] with inter-assay and intra-assay CVs lower than 5%. Total glutathione (GSH) was determined using the procedure described by Beutler et al. [30] and MDA by a spectrophotometric method [31] with inter-assay and intra-assay CVs lower than 5%.

### Clinical evaluation

According to the protocol of the study, the Beck Depression Inventory (BDI) has been evaluated by a modified questionnaire [32]. Anxiety has been gauged by the Beck Anxiety Inventory (BAI) designed by Beck et al. [33].

### Statistical procedures and size of the sample

According to the research design, the sample size formula has been employed for the randomized clinical trial, in which type 1 (α) and type 2 errors (β) have been 0.05, and 0.20 (power = 80%). One of the studies performed in this topic applied 0.80 as the SD and 0.64 as the changes in the mean (d) of HOMA-IR [7]. HOMA-IR chose to estimate sample size because it was the most important outcome in subjects with T2DM and CVD. Considering the power analysis, 25 subjects were required in each group. Upon the 20% drop-outs in each group, the size of the sample has been 30 subjects.

Kolmogorov-Smirnov statistic was employed to control data normality. Independent-sample *t*-test has been used for determining the difference in the anthropometric measures and dietary intake between the two groups. Multiple linear regression model was employed to evaluate the treatment impacts on the research outputs after setting for baseline values of the biochemical parameters. The effect size was provided as the mean difference with 95% confidence interval. Then, Pearson Chi-square test was used to compare categorical variables. *P*-values less than 0.05 were considered significant. Afterwards, SPSS18 (SPSS Inc.; Chicago, Illinois: USA) have been used for statistical analysis of the present trial.

## Results

**Fig. 1:**
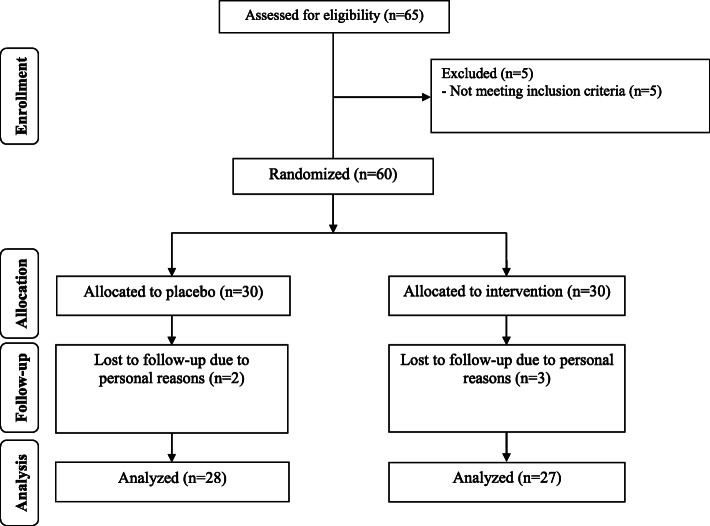
Summary of patient flow diagram

Fifty-five females [magnesium plus zinc (*n* = 27) and placebo (*n* = 28)] participated in the trial (Fig. 1). The compliance rate was high; both groups took > 90% of capsules during this trial. There was no adverse effects in the group of T2DM patients with CHD when taking magnesium plus zinc supplement.

There was no significant difference between the two groups regarding mean body height, weight, age, and body mass index (Table 1).

**Table 1:** General characteristics of study participants at baseline study

	Placebo group (*n* = 28)	Magnesium plus zinc group (*n* = 27)	*P*^a^
Age (y)	62.6 ± 10.8	61.7 ± 9.4	0.74
Height (cm)	159.5 ± 10.9	162.1 ± 7.8	0.32
Weight at study baseline (kg)	76.9 ± 12.6	81.3 ± 11.7	0.19
Weight at end-of-trial (kg)	77.0 ± 12.5	80.9 ± 11.4	0.23
Weight change (kg)	0.1 ± 1.2	−0.4 ± 1.0	0.12
BMI at study baseline (kg/m^2^)	30.2 ± 3.7	30.9 ± 3.8	0.47
BMI at end-of-trial (kg/m^2^)	30.2 ± 3.8	30.8 ± 3.7	0.59
BMI change (kg/m^2^)	0.03 ± 0.5	−0.1 ± 0.4	0.10

Data are means± SDs

^a^Obtained from independent *t*-test

Macronutrient and micronutrient ingestion as computed on the basis of the three-day food records did not differ significantly between magnesium plus zinc group and the controls (Supplementary file 1).

Magnesium and zinc co-supplementations significantly reduced FPG (β − 9.44 mg/dL, 95% CI, − 18.30, − 0.57; *P* = 0.03) and insulin levels (β − 1.37 μIU/mL, 95% CI, − 2.57, − 0.18; *P* = 0.02), and significantly increased the levels of HDL-cholesterol (β 2.09 mg/dL, 95% CI, 0.05, 4.13; *P* = 0.04) when compared with the placebo group (Table 2). Magnesium plus zinc taking was associated with a significant decrease in CRP (β − 0.85 mg/L, 95% CI, − 1.26, − 0.45; *P* < 0.001), and a significant increase in total nitrite (β 5.13 μmol/L, 95% CI, 1.85, 8.41; *P* = 0.003) and TAC (β 43.44 mmol/L, 95% CI, 3.39, 83.50; *P* = 0.03) when compared with the placebo group. Magnesium and zinc co-supplementation also significantly decreased BDI (β − 1.66; 95% CI, − 3.32, − 0.009; *P* = 0.04) and BAI scores (β − 1.30, 95% CI, − 2.43, − 0.16; *P* = 0.02) when compared with the placebo group. Magnesium and zinc co-supplementation did not have any significant effects on the other metabolic parameters when compared with the placebo group.

**Table 2:** The effect of combined magnesium and zinc supplementation on metabolic status in patients with T2DM and CHD

Variables	Placebo group (n = 28)		Magnesium and zinc group (n = 27)		Difference in outcome measures between magnesium and zinc treatment groups^a^	
	Baseline	Week 12	Baseline	Week 12	β (95% CI)	*P*^b^
Magnesium (mg/dL)	1.84 ± 0.22	1.83 ± 0.25	1.94 ± 0.21	2.08 ± 0.22	0.15 (0.08, 0.22)	< 0.001
Zinc (μg/dL)	93.3 ± 24.8	95.8 ± 25.9	98.1 ± 22.5	116.5 ± 21.5	16.21 (11.98, 20.44)	< 0.001
FPG (mg/dL)	123.7 ± 31.4	128.4 ± 32.7	119.2 ± 38.0	115.6 ± 28.7	−9.44 (−18.30, −0.57)	0.03
Insulin (μIU/mL)	13.8 ± 4.5	13.9 ± 4.4	12.9 ± 5.1	11.7 ± 4.9	−1.37 (−2.57, −0.18)	0.02
HOMA-IR	4.2 ± 1.8	4.2 ± 1.7	3.7 ± 1.7	3.4 ± 1.8	−0.36 (− 0.75, 0.02)	0.06
QUICKI	0.31 ± 0.02	0.31 ± 0.02	0.32 ± 0.02	0.32 ± 0.02	0.006 (−0.002, 0.01)	0.12
Triglycerides (mg/dL)	123.7 ± 50.7	124.9 ± 46.5	128.2 ± 55.7	134.6 ± 53.9	5.66 (−5.40, 16.73)	0.30
VLDL-cholesterol (mg/dL)	24.7 ± 10.1	24.9 ± 9.3	25.6 ± 11.1	26.9 ± 10.8	1.10 (−1.08, 3.34)	0.30
Total cholesterol (mg/dL)	147.3 ± 35.3	145.6 ± 31.0	137.1 ± 28.3	140.7 ± 32.5	3.72 (−5.26, 12.71)	0.41
LDL-cholesterol (mg/dL)	77.2 ± 29.5	76.6 ± 26.5	70.8 ± 22.4	71.6 ± 25.7	0.13 (−8.32, 8.59)	0.97
HDL-cholesterol (mg/dL)	45.3 ± 7.7	43.9 ± 7.0	40.6 ± 8.4	42.1 ± 8.2	2.09 (0.05, 4.13)	0.04
Total−/HDL-cholesterol ratio	3.3 ± 0.8	3.3 ± 0.7	3.4 ± 0.7	3.4 ± 0.8	−0.06 (−0.30, 0.17)	0.59
CRP (mg/L)	3.0 ± 1.0	3.2 ± 1.2	2.7 ± 1.7	2.1 ± 1.3	−0.85 (−1.26, − 0.45)	< 0.001
Total nitrite (μmol/L)	49.8 ± 6.1	48.7 ± 6.1	43.7 ± 5.3	50.1 ± 6.5	5.13 (1.85, 8.41)	0.003
TAC (mmol/L)	898.4 ± 168.4	894.9 ± 178.9	940.9 ± 107.2	976.8 ± 105.3	43.44 (3.39, 83.50)	0.03
GSH (μmol/L)	508.3 ± 72.9	524.9 ± 95.3	556.8 ± 92.9	578.2 ± 56.3	29.49 (−8.83, 67.82)	0.12
MDA (μmol/L)	2.2 ± 0.6	2.2 ± 0.5	1.9 ± 0.4	1.8 ± 0.4	−0.15 (−0.32, 0.007)	0.05
BDI score	19.7 ± 5.8	19.6 ± 5.7	21.7 ± 4.7	19.6 ± 4.9	−1.66 (−3.32, −0.009)	0.04
BAI score	15.7 ± 4.3	14.1 ± 4.8	17.0 ± 5.3	14.0 ± 5.1	−1.30 (−2.43, −0.16)	0.02

Data are mean ± SDs

*BDI* Beck Depression Inventory, *BAI* Beck Anxiety Inventory, *CHD* coronary heart disease, *FPG* fasting plasma glucose, *GSH* total glutathione, *HOMA-IR* homeostasis model of assessment-estimated insulin resistance, *CRP* C-reactive protein, *MDA* malondialdehyde, *QUICKI* quantitative insulin sensitivity check index, *T2DM* type 2 diabetes, *TAC* total antioxidant capacity

^a^”Outcome measures” refers to the change in values of measures of interest between baseline and week 12. β [difference in the mean outcome’s measures between treatment groups (magnesium and zinc group = 1 and placebo group = 0)]

^b^Obtained from multiple regression model (adjusted for baseline values of each biochemical variables)

## Discussion

This study demonstrated that magnesium plus zinc supplementation to subjects suffering from CHD and T2DM had beneficial effects on FPG, insulin, HDL-cholesterol, CRP, total nitrite, TAC, BDI and BAI scores. However, it must be kept in mind that although the difference in few variables including FPG and HDL-cholesterol in this study was statistically significant, it was not clinically significant. Long-term interventions with magnesium plus zinc supplementation might result in greater changes in FPG and HDL-cholesterol levels.

### Impact of magnesium plus zinc supplementation on glycemic control and lipoproteins

This study showed that magnesium plus zinc supplements in subjects with CHD and T2DM during 12 weeks caused a significant decrease in FPG and insulin levels, and significant increase in HDL-cholesterol, but it had no effect on the HOMA-IR, QUICKI, total cholesterol, triglycerides, VLDL-cholesterol and LDL-cholesterol levels. According to previous studies, chronic hyperglycemia and dyslipidemia due to increasing insulin resistance and inflammatory cytokines increase the risk of diabetic and atherogenic complications [34, 35]. Earlier, it was reported that nutritional supplements have the beneficial effects on metabolic profiles [8, 36]. A meta-analysis indicated that magnesium intake is associated with a significant improvement in FPG, triglycerides, HDL-cholesterol and LDL-cholesterol levels [37]. In a study on pregnant women with GDM who were taking as a supplement magnesium oxide (250 mg/day) during 6 weeks, a significant improvement in the FPG, insulin concentration, HOMA-IR, QUICKI, and triglycerides levels occurred, but it caused no change in other serum lipoproteins [7]. However, magnesium lactate in a dose of 360 mg per day during 12 weeks did not have significant effects on HbA1c, FPG, insulin levels, HOMA-IR and lipid profiles in normomagnesemic patients with T2DM [38]. One meta-analysis showed that supplementing zinc to patients with T2DM has been associated with a considerable decrease of FPG, HbA1c, total cholesterol, and a significant increase of HDL-cholesterol, but there has been no significant association with triglycerides [39]. Another study performed by Islam et al. showed that zinc supplementation (30 mg per day of zinc sulphate) during 6 months improved FPG, insulin resistance, insulin sensitivity and decreased triglycerides levels without causing any significant changes in LDL-cholesterol and HDL-cholesterol [10]. A combination of magnesium-zinc-calcium-vitamin D during a 12 week period has been associated with a significant decrease of HOMA-IR, insulin, triglycerides, total cholesterol, and VLDL-cholesterol and a significant increase in the QUICKI scores, but it has not been accompanied by changes in LDL-cholesterol, FPG, and HDL-cholesterol in the PCOS patients [14]. Hyper-insulinemia and insulin resistance have been related to hyperglycemia which is the main symptom of T2DM. Hyperglycemia affects the glycation of lipoproteins but has a lot of other unfavorable effects which cause accelerated atherosclerosis [40, 41]. Magnesium is one of the crucial cofactors in the enzymatic processes that require adenosine triphosphate and kinase, and therefore it plays an important role in glucose metabolic pathways [15, 42]. Magnesium also contributes to lipoprotein metabolism by modulating HMG-CoA reductase enzyme [16]. Zinc is involved in a range of functions including insulin receptor signal transduction, secretion and tissues/organelle distribution, and inhibition of protein tyrosine phosphatases [17, 43, 44]. Zinc may increase glucose transport into the cells by increasing phosphorylation of the β subunit of the insulin receptor and enhancing the activation of phosphatidylinositol 3 kinase and protein kinase B or Akt [45]. Furthermore, few zinc transporters (such as ZnT8) are important for the structure, secretion and compartmentalization of insulin in beta-cells of pancreas [46]. Zinc also stimulates IRAP molecule, which in turn enables the translocation of GLUT4 to the cell surface and enables the transport of glucose into the cell [47]. Zinc plays an important role in the stabilization of insulin hexamers and the pancreatic storage of insulin as well [43].

### Effects of magnesium plus zinc supplementation on oxidative stress and inflammation

This study showed a significant decrease of CRP and a significant increase of TAC and total nitrite as a result of combined magnesium and zinc supplementation to the patients with CHD and T2DM during a 12 weeks period. Oxidative stress and inflammation because of developing micro- and macrovascular complications are important risk factors for diabetes and diabetes-associated atherosclerosis [48]. Asemi et al. study indicated that magnesium supplement.

(250 mg/day of magnesium oxide) in pregnant women with GDM during 6 weeks significantly decreased hs-CRP, but there were no significant changes in TAC and GSH concentrations [7]. Nevertheless, magnesium supplement (magnesium oxide) in a dose of 250 mg per day during 8 weeks to overweight women did not cause any significant changes in inflammatory markers [49]. In another study, taking zinc supplement (30 mg per day of zinc sulphate) during 6 months did not cause any significant change in CRP in women with pre-diabetes [10]. However, magnesium-zinc-calcium-vitamin D supplementation for 12 weeks in women with PCOS was associated with a decrease in CRP levels [14]. Combined magnesium-zinc-calcium-vitamin D supplementation in another similar study caused a significant decrease in hs-CRP and MDA, and an increase in TAC without any significant changes in nitric oxide (NO) and GSH levels [50]. A recent study reported that combined magnesium and zinc supplementation (250 mg/day of magnesium oxide plus 220 mg/day zinc sulfate) to women with PCOS during 12 weeks improved hs-CRP and TAC although no significant effects were been seen on NO, MDA and GSH levels [13]. However, these discrepancy between studies may be due to differences in sampling method, study duration, age ranges, dosage of magnesium and zinc used, the characteristics of participants, differences between intervention and control groups, cross-over design or parallel design, and dietary intake of participants.

Magnesium is assumed to have anti-inflammatory properties caused by its antagonist effects to calcium which contributes to inflammation, transmembrane ion transport, and protein synthesis [21]. Magnesium also increases the production of NO and prostacyclins [22]. Zinc seems to have effects on hemostasis by influencing coagulation and platelet aggregation [23]. Zinc deficiency has impact on calcium channels and calcium uptake defects [24]. Also, second-messenger performance probably originates from unusual sulfhydryl redox states in the membrane channel protein having an impact on CVD [24]. Moreover, zinc may decrease inflammation and oxidative damage having effects on hemostasis by influencing coagulation and platelet aggregation [23], and reducing the activity of calcium channels [24]. Magnesium may have anti-inflammatory effects because of its antagonist effects to the calcium which contributes to inflammation, transmembrane ion transport, and protein synthesis [21] and increased production of prostacyclins [22].

### Effects of magnesium plus zinc supplementation on depression and anxiety

This study indicated that combined magnesium and zinc supplementation to patients with CHD and T2DM during 12 weeks improved BDI and BAI scores. The prevalence of depression and anxiety in CHD subjects is high and it is responsible for an increased risk of mortality influencing healthy lifestyle and increased motivation to stay healthy and compliance to therapy [51]. A study using food frequency questionnaire and general health questionnaire reported a reverse correlation between dietary magnesium intake and depression and anxiety [52]. A review suggested that zinc deficiency due to reduced zinc absorption and low intake of dietary zinc is prevalent in mood disorders and that zinc supplementation can have beneficial impacts [53, 54]. Nonetheless, Fard et al. showed that supplementation with 27 mg per day zinc sulfate or 320 mg/day magnesium sulfate did not improve postpartum anxiety and depressive symptoms after 8 weeks [55]. In another study by Nikseresht et al. administration of combined 30 mg/kg zinc chloride, 30 mg/kg magnesium chloride and 50 mg/kg thiamine-HCl in mice with postpartum depression symptoms improved these symptoms and anxiety-like behavior [56]. Zinc and magnesium act as cofactors and contribute significantly to synthesis and release of neurotransmitters and thereby can have antidepressant and anxiolytic effects [57]. For example, zinc and magnesium prevent binding of N-methyl-D-aspartate receptors to glutamate and may be associated with antidepressant and anxiolytic effects [58].

### Strength and study limitation

The present study has a number of strengths. The current study focused on novel questions using a randomized, double-blind, placebo-controlled trial. The findings of improved FPG, insulin, total nitrite, HDL-cholesterol, CRP, TAC levels, BDI and BAI score in the intervention group in the current study are interesting, but need to be confirmed in a larger study. Another strength of this study was the low dropout rate. This study has some limitations. The most important one is relatively small number of patients despite the fact that the power analysis showed that the number of participants is sufficient. In addition, due to funding limitations, gene expression related to insulin, lipid, oxidative damage and inflammation in patients with T2DM and CHD could not be evaluated.

## Conclusions

The combined magnesium and zinc supplementation during 12 weeks had beneficial effects on FPG, insulin, total nitrite, HDL-cholesterol, CRP, TAC levels, BDI and BAI score. However, it had no significant impact on other metabolic variables in patients with T2DM and CVD. This suggests that magnesium and zinc co-supplementation may be beneficial for patients with T2DM and CHD. Further research is needed on more patients and for longer periods to determine the safety of magnesium and zinc co-supplementation.

## Supplementary information

**Additional file 1: Supplemental file 1.** Dietary intakes of study participants throughout the study

## Data Availability

The primary data for this study is available from the corresponding author (Mohsen Taghizadeh) on reasonable request.
