# Supplementary material for: The effects of combined magnesium and zinc supplementation on metabolic status in patients with type 2 diabetes mellitus and coronary heart disease
Source: Lipids Health Dis. 2020 May 28;19:112. doi: 10.1186/s12944-020-01298-4 (PMC7257447; doi:10.1186/s12944-020-01298-4)
Supplement: Supplementary file 1 — Additional file 1: Supplemental file 1. Dietary intakes of study participants throughout the study [file 12944_2020_1298_MOESM1_ESM.docx]

**Supplemental file 1.** Dietary intakes of study participants throughout the study

|  | Placebo group  (n=28) | Magnesium plus zinc group (n=27) | *P*^1^ |
| --- | --- | --- | --- |
| Energy (kcal/d) | 2073±194 | 2009±254 | 0.29 |
| Carbohydrates (g/d) | 289.9±34.7 | 286.7±52.0 | 0.79 |
| Protein (g/d) | 74.0±12.1 | 72.6±16.4 | 0.72 |
| Fat (g/d) | 71.8±11.1 | 66.9±13.7 | 0.15 |
| SFAs (g/d) | 21.8±4.7 | 20.8±4.8 | 0.40 |
| PUFAs (g/d) | 21.9±4.7 | 21.3±5.7 | 0.63 |
| MUFAs (g/d) | 19.8±5.7 | 18.5±5.4 | 0.38 |
| Cholesterol (mg/d) | 180.2±71.0 | 169.5±99.2 | 0.64 |
| TDF (g/d) | 17.0±3.9 | 17.4±4.1 | 0.72 |
| Magnesium (mg/d) | 242.0±54.2 | 237.0±52.1 | 0.72 |
| Zinc (mg/d) | 9.3±1.8 | 9.0±2.5 | 0.57 |

Data are means± SDs.

^1^ Obtained from independent t test.

MUFAs, monounsaturated fatty acids; PUFAs, polyunsaturated fatty acids; SFAs, saturated fatty acids; TDF, total dietary fiber.
